# The triangle of anxiety, perfectionism, and academic procrastination: exploring the correlates in medical and dental students

**DOI:** 10.1186/s12909-024-05145-3

**Published:** 2024-02-23

**Authors:** Parvin Rezaei-Gazki, Mehran Ilaghi, Nahid Masoumian

**Affiliations:** 1 Department of Medical Education, Education Development Center, Kerman University of Medical Sciences, Kerman, Iran; 2 Institute of Neuropharmacology, Kerman Neuroscience Research Center, Kerman University of Medical Sciences, Kerman, Iran; 3 Hormozgan University of Medical Sciences, Bandar Abbas, Iran

**Keywords:** Anxiety, Perfectionism, Academic procrastination, Medical students, Dental students, Medical education

## Abstract

**Introduction:**

Academic procrastination is a common phenomenon among medical science students. This issue can negatively affect the students’ academic performance. The aim of this study was to investigate perfectionism and anxiety as potential predictors of academic procrastination in medical and dental students.

**Methods:**

This cross-sectional study was conducted among medical and dental students of a public medical sciences university in the south of Iran. Students were assessed using the procrastination assessment scale for students (PASS), Tehran multidimensional perfectionism scale (TMPS), and anxiety subscale of the general health questionnaire (GHQ). Predictors of academic procrastination were evaluated using multiple regression analysis with adjustments made for gender and academic semester.

**Results:**

A total of 176 medical and 79 dental students participated in the study. None of the perfectionism components were predictors of academic procrastination. However, anxiety was a significant predictor of academic procrastination in the total sample of students (standardized β = 0.404, *p* < 0.001), as well as dental (standardized β = 0.356, *p* < 0.001) and medical (standardized β = 0.478, *p* < 0.001) students. Anxiety and academic procrastination were both negatively correlated with students’ grade point averages.

**Conclusions:**

The findings of this study suggest that anxiety, as opposed to perfectionism, has a more significant influence on academic procrastination among medical and dental students. Interventions aimed at lowering anxiety may be beneficial for reducing academic procrastination, thereby enhancing the academic performance of the students.

## Introduction

The life of modern society is often filled with a variety of duties and assignments, most of which are accompanied by a set of deadlines and certain time limits. In a setting of constant tension and multitasking, it frequently occurs that there is a need or desire to put off performing some duties for a later time. This phenomenon of postponing tasks is generally referred to as procrastination and is defined as a *“voluntary, irrational postponement of the intended actions, despite the fact that this entails a negative effect on the individual”* [1].

Among different types of procrastination, academic procrastination is a widespread behavioral tendency to avoid beginning or completing necessary academic tasks even when the delay would have unfavorable outcomes [2]. This phenomenon is quite common among university students, particularly those majoring in medical sciences, and is believed to be a significant concern that negatively affects learning and academic performance [3–5].

From a psychological point of view, various behavioral, emotional, and cognitive components might contribute to the development of procrastination in an individual. These encompass a variety of factors, including fear of failure, low self-esteem, self-regulation, neuroticism, and inability to make decisions independently [6–8]. One might also subconsciously use procrastination as a defensive mechanism when accomplishing a task is perceived as threatening [9]. In an attempt to better elucidate the correlates of procrastination, a bulk of recent evidence has suggested that other factors, including perfectionism and anxiety, may be involved in academic procrastination [9, 10].

Perfectionism is generally defined as possessing high-performance standards accompanied by tendencies for a self-critical evaluation while pursuing flawlessness [11]. People demonstrating excessive perfectionistic tendencies seek to avoid conditions where they have to meet unrealistically high standards [12]. As a result, some aspects of perfectionism might have pivotal roles in preserving academic procrastination. This has been suggested by several studies, including a study by Jadidi et al., which demonstrated that higher perfectionism was associated with higher levels of academic procrastination [13]. Medical and dental students are frequently viewed as perfectionists. However, to determine the extent to which perfectionism may contribute to academic procrastination, additional research is required.

Anxiety is considered another potential contributing factor to academic procrastination. Based on Freudian psychoanalysis, procrastination is basically a result of anxiety [14]. According to this concept, anxiety is regarded as a sign of repressed unconscious desires of the “self”. The Freudian notions of dynamic defenses and task avoidance suggest that tasks that are not accomplished are avoided since they are threatening to the ego [14]. This concept has also been reinforced by some studies among college students, where anxiety proved to be associated with procrastination [15, 16].

While procrastination can certainly manifest as part of broader anxiety-related dysfunction, students who struggle with procrastination in a perfectionistic elite environment may not outwardly display anxiety or impeded functioning. Therefore, understanding factors predicting procrastination itself could aid early intervention. The consequences of academic procrastination among students of medical sciences, who are likely to bear considerable responsibilities in their impending professional careers, could be catastrophic. Therefore, the conceptual foundations of academic procrastination may need to be further addressed in order to offer insight into effective preventive strategies. In light of this, we aimed to investigate the interrelationship between perfectionism and anxiety as potentially related factors of academic procrastination in students majoring in medical sciences. Additionally, due to different educational contexts within various fields of medical sciences, we expanded our scope to examine whether these correlates are different among students of two distinct majors of medical sciences, namely dentistry and medicine.

## Methods

### Study design and setting

This was a quantitative exploratory cross-sectional study aiming to investigate perfectionism and anxiety as potential predictors of academic procrastination among medical and dental students.

The study was carried out at Hormozgan University of Medical Sciences (HUMS), one of the major medical universities located in the south of Iran, with more than 3600 students studying in seven faculties. The study was performed on medical and dental students in the 2017–2018 academic year.

### Participants

Inclusion criteria for the enrollment of students were as follows: medical and dental students who had been studying at HUMS in the 2017–2018 academic year. Exclusion criteria were as follows: students who did not consent to participate, as well as students who subjectively reported a clinically diagnosed anxiety disorder or were under treatment for anxiety disorders.

### Sampling

According to the total number of students registered in the two majors at the time of the study, the minimum required sample size was determined to be 300 based on Cochran’s sampling formula and considering a 5% margin of error.

To guarantee that a minimum of participants in each major were enrolled in the study, a quota sampling technique was utilized based on the number of registered students in the medical and dental programs at the time of participant recruitment. Specifically, the total sample was allocated proportionally between majors to match the distribution of enrolled students, with nearly 70% medical and 30% dental students according to the total registered students. This ensured adequate representation within each subgroup for comparison purposes. In each major, students were enrolled using convenience sampling. Incomplete surveys were finally excluded from analysis; however, a post-hoc power calculation with the remaining study sample demonstrated a study power > 0.95 for the analyses made within the total sample as well as each discipline, thereby indicating a sufficient sample size.

Students were recruited through direct contact at the dormitories, campus store, clinical shift breaks, lecture breaks, or the conclusion of morning reports. Participation was voluntary, and students were enrolled upon informed consent.

### Procedure and Instruments

To collect data, three valid and reliable questionnaires were used as follows:

#### Procrastination assessment scale for students (PASS)

This scale was originally introduced by Solomon and Rothblum as a measure of academic procrastination [17]. The Persian version of this questionnaire has been used in various studies in Iran, and its validity and reliability have been reported at acceptable levels [3, 18]. In line with previous studies, in this study, the validated 27-item Persian scale was used, which addresses exam preparation (8 items), task accomplishment (11 items), and final term assignments (8 items) and is scored based on a 5-point Likert scale (1 = never to 5 = always) [6].

#### Tehran multidimensional perfectionism scale (TMPS)

This scale was first developed and validated in Persian by Besharat [19]. The questionnaire contains 30 items in three dimensions: self-oriented perfectionism (SOP), other-oriented perfectionism (OOP), and socially-prescribed perfectionism (SPP), which is scored using a 5-point Likert scale (1 = strongly disagree to 5 = strongly agree) [19]. According to Hewitt and Flett, SOP involves pursuing irrational high standards for oneself, while OOP refers to similar behavior directed toward others instead of the self, and SPP comprises the belief that others have perfectionistic expectations and motives for themselves [20].

#### Anxiety/insomnia subscale of general health questionnaire-28 (GHQ-28)

The GHQ-28 has been developed and introduced as a screening tool to detect those likely to have or to be at risk of developing psychiatric disorders. This scale is a 28-item measure of common mental health problems, including somatic symptoms, anxiety/insomnia, social, and severe depression. The Persian version of GHQ-28 has shown desirable validity and reliability [21]. In this study, the anxiety/insomnia subscale was used, which includes seven items that are scored based on a 4-point Likert scale (0 = not at all to 3 = much more than usual).

The questionnaires were distributed in person, the objectives of the study were clearly outlined, and informed consent was obtained from all participants while the confidentiality of their identity was guaranteed.

### Statistical analysis

Data were analyzed using the Statistical Package for the Social Sciences (SPSS) software (version 22.0. SPSS, Inc., Chicago, IL, USA). The categorical variables were described as frequency and percentage, and the continuous variables as mean (± SD). Linear regression analysis was performed where the score of academic procrastination was considered as the dependent variable and potential variables, including the scores of anxiety, SOP, OOP, and SPP as independent variables. All models were adjusted for the gender and academic semester of students. Collinearity diagnostics were performed using variance inflation factors (VIFs) for each independent variable. A *p*-value less than 0.05 was used to denote statistical significance.

## Results

Of the total 300 distributed questionnaires, 45 were excluded due to incomplete responses, and a total of 255 were finally included in the study. A total of 176 participants (69.0%) were medical students, while 79 (31.0%) were dental students. Most participants were female (65.5%), which constituted the majority of the participants in both medicine (71%) and dentistry (53.2%) discipline. The grade point average (GPA) of students was 15.7 ± 1.2. Table 1 demonstrates the demographic and educational characteristics of the participants (Table 1).

**Table 1:** Demographic and educational characteristics of students

Variables		N(%)
**Major**	Medicine	176 (69.0)
	Dentistry	79 (31.0)
**Gender**	Male	88 (34.5)
	Female	167 (65.5)
		**Mean (± SD)**
**GPA**		15.7 (± 1.2)

Based on the results obtained from PASS, TMPS, and the anxiety sub-scale of GHQ-28, the scores of students on academic procrastination, perfectionism, and anxiety are presented in Table 2 (Table 2).

**Table 2:** Scores obtained on academic procrastination, perfectionism, and anxiety according to PASS, TMPS, and anxiety sub-scale of GHQ-28 questionnaires

Scale		Minimum score obtained	Maximum score obtained	Mean score	Standard deviation
**Academic procrastination**		42.00	113.00	82.47	11.61
**Perfectionism**	**SOP**	18.00	46.00	33.61	4.35
	**OOP**	21.00	45.00	34.40	4.29
	**SPP**	15.00	47.00	41.84	4.25
**Anxiety**		0.00	21.00	7.14	4.30

In order to identify the predictors of academic procrastination, potential variables, including the score of different components of perfectionism and anxiety score were entered into a multiple linear regression model. Models were adjusted for the gender and academic semester of the students. The analysis was performed in the total sample as well as distinct subgroups of medical or dental students. Results demonstrated that none of the perfectionism components were significant predictors of academic procrastination. However, anxiety was a significant predictor of academic procrastination in the total sample of students (standardized β = 0.404, *p* < 0.001) as well as in the subgroups of medical (standardized β = 0.356, *p* < 0.001) and dental (standardized β = 0.478, *p* < 0.001) students (Table 3).

**Table 3:** Multiple linear regression model with academic procrastination score as dependent variable. The analysis is performed in the total sample as well as the subgroup of students based on academic majors

Independent variable	All (*n* = 255)				Medical Students (*n* = 176)				Dental Students (*n* = 79)			
	β	SE	Std β	***p***	β	SE	Std β	***p***	β	SE	Std β	***p***
**Anxiety score**	1.091	0.160	0.404	< 0.001	0.977	0.205	0.356	< 0.001	1.254	0.277	0.478	< 0.001
**SOP score**	-0.265	0.187	-0.099	0.157	-0.279	0.208	-0.117	0.181	-0.208	0.429	-0.061	0.630
**OOP score**	-0.162	0.188	-0.060	0.392	-0.143	0.215	-0.057	0.507	-0.266	0.400	-0.084	0.508
**SPP score**	-0.019	0.198	-0.007	0.922	-0.045	0.225	-0.018	0.842	0.199	0.432	0.062	0.647

Models are adjusted for gender and academic semester of students. **β**: Unstandardized β coefficient; **SE**: Standard Error; **Std β**: Standardized β coefficient

As a secondary objective of our study, we investigated the correlation of perfectionism, anxiety, and academic procrastination scores with the GPA of students. The GPA showed a significant negative correlation with academic procrastination score (*r*=-0.314, *p* < 0.001), and a significant negative correlation with anxiety score (*r*=-0.152, *p* = 0.015). However, no significant relationship was observed between GPA and any components of perfectionism (Table 4).

**Table 4:** Correlation between GPA, perfectionism, anxiety, and academic procrastination scores

		SOP Score	OOP Score	SPP Score	Anxiety Score	Academic procrastination Score
**GPA**	Correlation coefficient	0.082	0.010	0.015	-0.152	-0.314
	*p*-value	0.194	0.876	0.814	0.015	< 0.001

## Discussion

While there is no prevailing theory to explain procrastination in medical and dental students, common themes have emerged in the literature. Given that academic procrastination is frequently viewed as a serious personal and situational problem, the purpose of this study was to provide preliminary evidence for its correlates in order to develop a basis for interventions that can aid instructors in higher education communities in assisting students in preventing and reducing procrastination tendencies. The primary findings of our study demonstrated that perfectionism was not a significant predictor of academic procrastination among medical and dental students, although anxiety proved to be a major predictor.

Various components of perfectionism could be both positive and negative predictors of academic procrastination. Cho and Lee demonstrated that SOP could be a protective factor against academic procrastination, concluding that those students who strive to achieve high personal standards are less likely to procrastinate [2]. Similarly, in a meta-analysis by Xie et al., it was reported that perfectionistic striving (i.e., high performance standards and a self-oriented striving for perfection) was negatively linked to procrastination, whereas perfectionistic concerns (i.e., a feeling of a discrepancy between expectations and results, concern over a mistake, and doubts about actions) were positively associated with procrastination [22].

Unlike the above studies, the findings of our study failed to show a significant association between either of the perfectionism components and academic procrastination. Although the inconsistency between studies might generally be due to different measures, constructs, and definitions of perfectionism, one explanation for our findings might be attributed to the nature of the studied population. Considering the fact that dentistry and medicine are generally considered the top university majors, particularly in the Iranian context, we hypothesize that after succeeding in enrolling in these majors, students tend to set high performance standards in order to value their endeavors and therefore adopt less maladaptive perfectionism. As a result, despite the potential negative consequences of perfectionism, this has not led to higher academic procrastination. Findings of a study on non-medical students in Iran showed that although the scores on different dimensions of perfectionism were relatively lower than what we observed among medical/dentistry students, maladaptive perfectionism was associated with procrastination [23]. Furthermore, our hypothesis is reinforced by previous studies that reported maladaptive perfectionism to be lower in medical students compared to the general population [24].

Our results further revealed that anxiety is a significant predictor of academic procrastination in both majors. This finding highlights that in students majoring in medical sciences, anxiety appears to be a more important factor than perfectionism in the development of academic procrastination. Accordingly, considerable evidence indicates that students majoring in medical sciences are confronted with several sources of anxiety [25, 26]. Some reports even indicate that anxiety symptoms are more prevalent among medical students than non-medical students [27, 28]. The anxiety scores of students in our study are also comparable to findings from other studies on Iranian medical students, which suggest a high prevalence of anxiety disorders among this population [29]. This could be a result of numerous clinical shifts, the multiplicity of assessments, and an intensive curriculum. In addition, these students often bear the responsibility of patient-care outcomes, which adds to the burden of several challenges in performing procedures and maintaining communication with patients.

Our findings support Freud’s psychoanalytic theory, indicating that tasks might be avoided since they are threatening to the ego, and procrastination is principally an outcome of anxiety [14]. On this basis, several authors have similarly provided explanations about the link between anxiety and procrastination. For instance, Hobfoll has pointed out the appraisal anxiety-avoidance model to explain the relationship between anxiety and procrastination. According to this paradigm, if individuals perceive a situation as a challenge that they cannot handle, they are likely to attempt to flee the circumstance [30]. Likewise, Ariani and Susilo revealed that test anxiety greatly influenced procrastination in a direct manner so that people who are anxious about their task may engage in a different activity as a means of relieving their existing stress [31]. We, however, argue that this association can be reciprocal such as where anxiety is the outcome of procrastination rather than merely the source. This is well-described by Wang, who found that academic procrastination leads to an increase in test anxiety [32]. This is foreseeable as the students who more frequently postpone tasks may end up with difficulties managing their assignments and test, which contributes to additional levels of anxiety.

Expectedly, our findings demonstrated that academic procrastination and anxiety were negatively correlated with students’ GPA. This is in line with previous studies indicating that academic procrastination could result in worse academic performance [33]. Accordingly, in a meta-analysis study, Kim and Seo reported that GPA, assignment grade, quiz score, and course grade were all negatively associated with procrastination [34]. The time pressure resulting from procrastination might be a contributing factor to the reduction of accuracy and punctuality which might negatively impact performance [35]. On the other hand, anxiety has been proposed as a significant contributor to academic performance, especially in medical and dental students. Mihăilescuet al. demonstrated that anxiety is inversely correlated with academic performance in medical students [36]. The same findings have been observed in dental students [37]. These findings highlight practical implications for interventions regarding academic procrastination and anxiety in dental and medical schools. This could involve either strengthening student support systems aiming to provide aid to students in need of help or improving students’ motivation and engagement in study activities. Future research is needed to develop and evaluate interventions aiming to reduce academic procrastination and anxiety among students majoring in medical sciences.

There were several limitations to the present study. First, this was a single-center study. Caution must be made while generalizing the results to students of other institutions with different contextual backgrounds. Second, we used a clinical scale for anxiety assessment. However, other components of anxiety, including test anxiety, might be correlated with academic procrastination as well. Therefore, utilizing other measures of anxiety could provide further details about the interaction between anxiety and academic procrastination. Third, the interrelationship of perfectionism, anxiety, and academic procrastination might be paved with other related factors that are not considered in the current study. For instance, depressive disorders as well as personality traits might be involved as potentially associated factors which were not independently investigated in this study due to limitations in the number of questionnaires distributed. Therefore, future research should address other potential factors that could moderate these relationships.

## Conclusions

Overall, building on the findings of this study, we propose that anxiety, as opposed to perfectionism, has a more significant influence on academic procrastination among dental and medical students. We suggest that interventions aimed at lowering anxiety may be beneficial for reducing academic procrastination, consequently enhancing the academic performance of these students.

## Data Availability

The data analyzed during the current study are available from the corresponding author upon reasonable request.
